# Supplementary material for: Splice-Junction-Based Mapping of Alternative Isoforms in the Human Proteome
Source: Cell Rep. Author manuscript; Available in PMC 2020 Jan 15. (PMC6961840; doi:10.1016/j.celrep.2019.11.026)
Supplement: 2 [file NIHMS1546469-supplement-2.zip › DF1/PXD006675/VentricularSeptum/VentricularSeptum_11_TTN_ASNEYGSVSCTATLTVTEPPR.pdf]

A

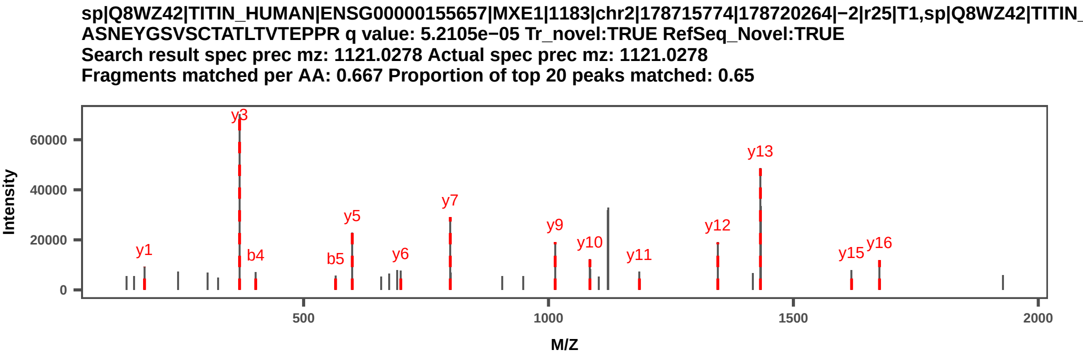

sp|Q8WZ42|TITIN_HUMAN|ENSG00000155657|MXE1|1183|chr2|178715774|178720264|−2|r25|T1,sp|Q8WZ42|TITIN_
ASNEYGSVSCTATLTVTEPPR q value: 5.2105e−05 Tr_novel:TRUE RefSeq_Novel:TRUE
Search result spec prec mz: 1121.0278 Actual spec prec mz: 1121.0278
Fragments matched per AA: 0.667 Proportion of top 20 peaks matched: 0.65
Intensity
M/Z
y1
y3
b4
b5
y5
y6
y7
y9
y10
y11
y12
y13
y15
y16

B

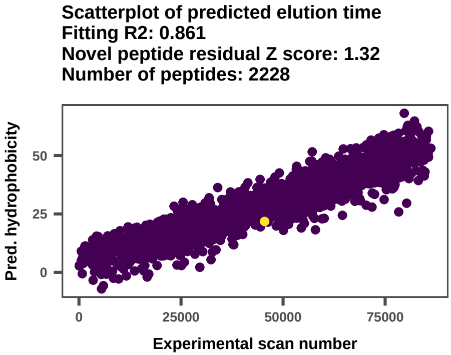

Scatterplot of predicted elution time
Fitting R2: 0.861
Novel peptide residual Z score: 1.32
Number of peptides: 2228
Pred. hydrophobicity
Experimental scan number

C

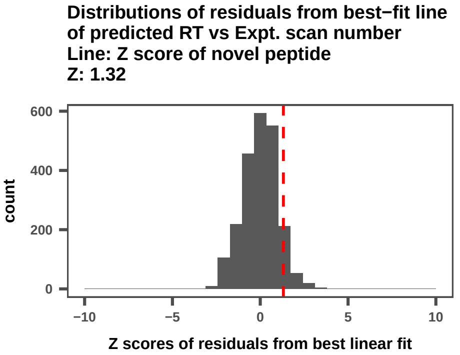

Distributions of residuals from best−fit line
of predicted RT vs Expt. scan number
Line: Z score of novel peptide
Z: 1.32
count
Z scores of residuals from best linear fit
